# Constant false alarm rate detection of pipeline leakage based on acoustic sensors

**DOI:** 10.1038/s41598-023-41177-3

**Published:** 2023-08-29

**Authors:** Guorui An, Zuheng Huang, Yanbing Li

**Affiliations:** School of Electronic and Information Engineering, Beijing Jiaotong University, Beijing, 100044 China

**Keywords:** Electrical and electronic engineering, Energy infrastructure

## Abstract

During the transportation of oil and gas pipelines, there are many potential factors that can lead to pipeline leakage with serious consequences, making automatic and real-time pipeline leakage detection urgent. In response to the inconvenience of manual detection, constant false alarm rate (CFAR) detection technique in radar target detection theory is introduced for pipeline leakage detection based on acoustic signals. In this paper, an automatic pipeline leakage detection algorithm based on an improved CFAR detector is proposed. The improved CFAR detection is executed after pre-processing the acoustic signals so as to adaptively set the detection threshold to achieve the purpose of automatic detection of pipeline leakage incidents. A simulated leakage test of a real pipeline is used for validation, and the proposed method achieves detection accuracies of 84.6%, 97.7%, and 98% for different leakage diameter settings, i.e., 5 mm, 7 mm, and 10 mm leak hole diameters, respectively, with an overall accuracy of 94.1%, while the false alarm rates are 3.3%, 0.7%, and 0, respectively, as well as an overall of 1.2%. The results of experimental data based on real scenarios demonstrate the effectiveness of the proposed method.

## Introduction

Energy transportation is the basis of modern industrialized societies. Connecting energy origins to densely populated consumer markets through energy transportation pipelines^1, 2^ not only reduces the imbalance between regions, but also reduces emissions in industrialized regions. For example, China’s West-to-East Gas Transmission Project delivers abundant natural gas resources from the west to the resource-poor east to release the contradiction of uneven resource distribution and help improve the fragile environment in the industrialized east^3^. The first two phases of the West-to-East gas pipeline network currently in operation cover a total distance of nearly 13,000 km. According to the design^4, 5^, the gas transmission capacity is 12 billion cubic meters in Phase I and 30 billion cubic meters in Phase II. In the face of energy transportation of such scale and length, safe operation is crucial and a major challenge. One of the issues that threatens the safe operation of energy transportation is pipeline leakage. Therefore, pipeline leak detection technology is an important way to ensure the safety of energy pipeline transportation.

Due to the complex terrain and climatic conditions, pipeline laying patterns are diverse^6^. Complex construction patterns also have more potential factors that lead to leaks. Therefore, leak detection requires an algorithmic model that can detect in real time and dynamically, without the interference of complex environments. Most of the earlier pipeline leak detection relied on manual detection, which greatly reduced the efficiency of detection. Therefore, an automated detection technology is necessary. In addition, it is possible to improve the accuracy of leak detection with the assistance of smart sensors^7^.

Several studies have been presented to analyze leak signals from oil and gas pipelines. The main focus in these studies was on acoustic sensor signals. Fuchs and Riehle demonstrated the feasibility of using acoustic fields for leak detection, which have been widely used for leak measurements^8^. Hou and Zhang proposed the use of the negative pressure wave principle for leak signal detection^9^. Wan^10^, Gao^11^ and Mostafapour^12^ also proposed to connect to two or more sensors to collect data for analysis. The analysis results show that the leakage signal is highly attenuated by the acoustic energy in the pipeline, and the attenuation is particularly pronounced in the high frequency component. Most of these findings are presented in terms of acoustic model characteristics. They clarify many of the main physical properties of the leak signal and the methods for capturing and analyzing it. However, they only emphasized the methods that rely mainly on manual supervision. Very little research has been done on detection algorithm models.

Based on the above analysis, this paper focuses on the study of automatic algorithms used for pipeline leak detection. An algorithmic model capable of automatically detecting pipeline leaks will be proposed. It will replace the previous manual monitoring or fixed-threshold detection methods, whose thresholds can be dynamically adjusted in real time according to the amplitude of the noise level, thus achieving a balance between false alarm probability and detection probability. To achieve this goal, the constant false alarm rate detector^13^ in radar detection theory is used for the real-time detection of pipeline leakage signals. Constant false alarm rate (CFAR) detector is an adaptive threshold method that detects the target with a constant false alarm probability level, which eliminates the problem of threshold failure due to noise level variations. Its real-time threshold adjustment property makes it an effective method for automatic detection and facilitates real-time implementation with less computation compared to machine learning based methods^14–16^. To the best of our knowledge, this is the first time that the idea of CFAR detection is applied to pipeline leakage detection.

The rest of the paper is organized as follows. “Pipeline leakage acoustic signal model” models the acoustic signatures of pipeline leaks. Then an automatic leak detection method based on CFAR is presented in “Pipeline leak detection based on constant false alarm rate detector”. Real-world experiment based results are shown and discussed in “Experimental results and discussion”. Finally, “Conclusion” concludes this paper.

## Pipeline leakage acoustic signal model

For pipeline leakage events, there are two types of situations that may be encountered: normal operating situation and leakage situation. The acoustic signals for each of the two cases will be modeled as follows.

### Signal model in normal operating situation

When no leakage occurs, the pipeline transport is in normal working condition, at this time the acoustic sensor will not receive abnormal signals, and the signal in this case can be modeled as Gaussian white noise as follows:

1 $$\begin{aligned} n(t)=\frac{1}{\sqrt{2 \pi } \sigma } e^{-\frac{(s-\mu )^2}{2 \sigma ^2}},-\infty<x<\infty \end{aligned}$$

where *t* is the time, *s* is the signal amplitude at time *t*, $\mu$ is the expectation of the noise signal, and $\sigma ^2$ is the variance of the noise signal.

In practical scenarios, the noise acquired by acoustic sensors mainly comes from thermal noise in circuit devices. Meanwhile, there may be some interference terms, such as electromagnetic interference or operating conditions variations of the transport pump. This makes the mean value of the noise may not be zero. That is, the noise signal is superimposed on some constant term. In addition, if the interference term may change with time, the noise signal will also have a trend term. In this case, the signal can be modeled as

2 $$\begin{aligned} n(t)=\frac{1}{\sqrt{2 \pi } \sigma } e^{-\frac{(s-\mu (t))^2}{2 \sigma ^2}},-\infty<x<\infty \end{aligned}$$

where $\mu (t)$ is the trend term which can change with time.

### Signal model in leakage situation

In general, the unstable motion of the fluid or gas during a pipeline leak causes the pipeline near the leak point to vibrate, which in turn causes oscillations in the acoustic signal. In this case, the oscillation of the signal can be summarized as follows:

1. The amplitude of the signal envelope follows an exponential decay.
2. Most of the amplitude and energy are concentrated in the transient phase of vibration.

The model of acoustic wave propagation in an ideal fluid medium in a pipeline is^17, 18^

3 $$\begin{aligned} \frac{\partial ^{2} s_{0}}{\partial x^{2}}=\frac{1}{c_{0}^{2}} \frac{\partial ^{2} s_{0}}{\partial t^{2}}, \end{aligned}$$

where *x* is the direction of propagation, $s_{0}$ is the sound pressure, and *c* is the speed of sound in air. The expression for the acoustic pressure solution of (3) with respect to plane waves in the pipeline is

4 $$\begin{aligned} s_{0}(t)=A_{m n} \cos \left( m \theta -\varphi _{m}\right) J_{m}\left( k_{m n} r\right) \textrm{e}^{\textrm{j}\left( \omega t-k_{x} x\right) }, \end{aligned}$$

where $A_{m n}$ is the sound pressure amplitude, $\omega$ is the angular frequency, and $k_{x}$ is the wave number, the $J_{m}\left( k_{m n} r\right)$ represent m-order column Bessel function with the argument of $k_{m n}r$, *r* and $\theta$ are the radial coordinates and the polar angle in the cylindrical coordinate system, respectively. Therefore, the main component of the acoustic signal model is the product of the exponential decay function and the cosine function.

Considering the noise effect, the model can be expressed as

5 $$\begin{aligned} s_{0}(t)=A_{m n} \cos \left( m \theta -\varphi _{m}\right) J_{m}\left( k_{m n} r\right) \textrm{e}^{\textrm{j}\left( \omega t-k_{x} x\right) }+n(t) \end{aligned}$$

In addition, in the process of pipeline transportation, due to changes in working conditions or the pump’s own parameters. There is also a slow-varying trend term in the acoustic signal. Therefore the final signal model is a combination of acoustic signal, noise and trend term. As shown below.

6 $$\begin{aligned} s_{0}(t)=A_{m n} \cos \left( m \theta -\varphi _{m}\right) J_{m}\left( k_{m n} r\right) \textrm{e}^{\textrm{j}\left( \omega t-k_{x} x\right) }+\beta t+n(t) \end{aligned}$$

where $\beta t$ is slow change trend term.

## Pipeline leak detection based on constant false alarm rate detector

### Preprocessing of acoustic signals

As mentioned above, the detecting equipment will encounter the environment of pump condition changes and electromagnetic interference during operation. This causes the signal to have a slow-varying trend term. Also, thermal noise exists in the sensor circuit. These factors can lead to false alarms or missed alarms during the detection process and cause interference in the detection of the signal. Therefore, preprocessing of the acoustic signal is required.

#### Data outlier elimination

In the process of signal acquisition by the sensor, there may be a moment of data loss due to the instability of the network environment during the transmission process, generating some abnormal data. When the data loss occurs, the abnormal signal usually produces a mutation point. In order to prevent this abnormal signal from being misjudged as a leak incident, special numerical processing of this abnormal signal is required to avoid the impact on the detection results.

Since outliers in a signal are usually characterized by randomness and sparse distribution. Median filtering can be used to process the outliers. The median filtering is realized as follows.

7 $$\begin{aligned} s_{m}(t)={\text {med}}\left[ {\text {sort}}\left[ s_{o}(t-n) \times w(n)\right] \right] , \end{aligned}$$

where *t* denotes the time, $s_{o}(t)$ is the original signal, ${\text {sort}}$ is the sorting function, ${\text {med}}$ is the median taking operation, and *w*(*n*) is a moving window defined as follows,

8 $$\begin{aligned} w(n)={\left\{ \begin{array}{ll} 1, &{} 1\le n\le N \\ nan, &{} else \end{array}\right. }, \end{aligned}$$

where *N* is the window length, and *nan* represents a non-existent value.

#### Data de-trending

In the process of media transmission in the pipeline, there may be a trend of slow change in the signal due to the change of working conditions, which makes the detection threshold may also fluctuate, resulting in false alarms or missed detections. Thus the trend of the signal need to be removed before leakage detection. By using the moving window difference, the signal trend can be eliminated. The moving window difference is realized as follows.

9 $$\begin{aligned} s_{d}(t)=s_{m} (t)-s_{m}(t-N), \end{aligned}$$

where *N* is the moving window length.

#### Data denoising

During the collection of acoustic signals, thermal noise in the sensor circuit is also recorded along with the acoustic waves. Therefore, after cleaning the data outliers and removing the trend components, the signal also needs to be processed for noise reduction to further improve the signal-to-noise ratio (SNR) and facilitate subsequent leak incident detection. The noise in the signal can be suppressed by moving mean. The implementation process is as follows.

10 $$\begin{aligned} s(t)=\frac{1}{N} { \sum _{n=0}^{N}}\left[ s_{d}(t-n)*w(n) \right] . \end{aligned}$$

### Introduction of CFAR detector

After performing signal preprocessing, the power signal can be obtained as follows and used as input for a CFAR detector:

11 $$\begin{aligned} s_{p}(t)=s^2(t). \end{aligned}$$

The CFAR detector is a technique for detecting a target in noise signals while maintaining a constant probability of false alarms. It has been widely used in radar target detection^19^. Compared with the detection technique with fixed threshold, whose performance degrades when the signal noise level changes, it can maintain better detection performance when the noise level changes due to the adaptive nature of its detection threshold. Therefore, the CFAR will be used in this paper to detect leakage events in acoustic signals.

In radar target detection, the probability of presence of a target under certain conditions is often unknown, nor is the loss caused by a missed detection. Therefore, the Neyman–Pearson criterion is often used in target detection, i.e., given the probability of false alarm condition to maximize the detection probability. To achieve this goal, two hypotheses are defined as follows,

12 $$\begin{aligned}{} & H_{0}: s_{p}(t)=\varepsilon (t) \\&H_{1}: s_{p}(t)=s_{tar}(t)+\varepsilon (t) ,\end{aligned}$$

where $H_{0}$ hypothesis indicates that there is only Gaussian white noise in the current signal and no target is present. $H_{1}$ hypothesis indicates the presence of a target $s_{tar}$ in the current signal, and the signal consists of a target plus Gaussian white noise.

**Figure 1:**
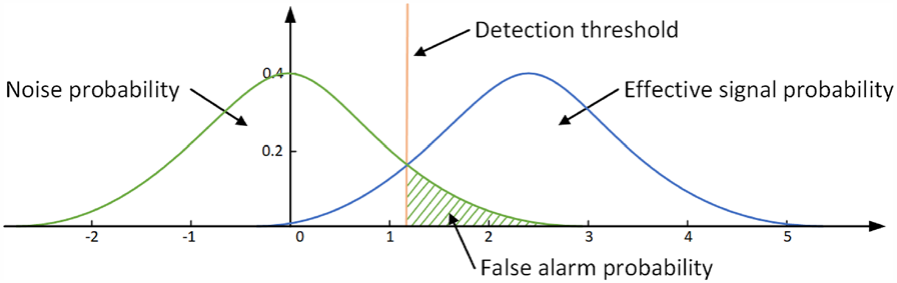
Schematic diagram of the probability distribution of CFAR detection.

When the $H_{0}$ hypothesis holds, i.e., only noise is present, the probability density function of the received signal is

13 $$\begin{aligned} p\left( s_{p} \mid H_{0}\right) =\frac{1}{\sqrt{2 \pi }} \exp \left( -\frac{s_{p}^{2}}{2}\right) , \end{aligned}$$

and when the $H_{1}$ hypothesis holds, i.e., the target and the noise exist simultaneously, the probability density function of the received signal is

14 $$\begin{aligned} p\left( s_{p} \mid H_{1}\right) =\frac{1}{\sqrt{2 \pi }} \exp \left( -\frac{(s_{p}-s_{tar})^{2}}{2}\right) , \end{aligned}$$

then the determination criterion for target detection is

15 $$\begin{aligned}{}&\frac{p\left( s_{p} \mid H_{1}\right) }{p\left( s_{p} \mid H_{0}\right) }>\Lambda _{0}, \quad \text {the verdict is } \textrm{H}_{1} \\&\frac{p\left( s_{p} \mid H_{1}\right) }{p\left( s_{p} \mid H_{0}\right) }<\Lambda _{0}, \quad \text {the verdict is }\textrm{H}_{0}, \end{aligned}$$

where, the likelihood ratio threshold $\Lambda _{0}$ is a variable proportional to the detection threshold $V_{T}$. Under hypothesis $H_{0}$, the probability of false alarm due to noise level exceeding the detecting threshold $V_{T}$ is expressed as

16 $$\begin{aligned} \begin{aligned} P_{fa}&=P\left( D_{1} \mid H_{0}\right) \\&=\int _{V_{T}}^{+\infty } p\left( s_{p} \mid H_{0}\right) d x, \end{aligned} \end{aligned}$$

where $D_{1}$ represents the event that the target is detected, $V_{T}$ is determined by the assumption that $P_{fa}$ is a constant as shown in Fig. 1. It can be seen from Fig. 1, the green curve is the probability density function in $H_{0}$ hypothesis, and the blue curve is the probability density function in $H_{1}$ hypothesis. Then the false alarm probability is equal to the area of the green curve in the right part of the detection threshold. When the area of this part is constant, i.e., the false alarm probability is constant, the detection threshold $V_{T}$ can be determined. Then the likelihood ratio threshold $\Lambda _{0}$ can be obtained by the normal inverse cumulative distribution function according to $V_{T}$.

In this case, the detection probability can be obtained as

17 $$\begin{aligned} \begin{aligned} P_{d}&=P\left( D_{1} \mid H_{1}\right) \\&=\int _{V_{T}}^{+\infty } p\left( s_{p} \mid H_{1}\right) d x. \end{aligned} \end{aligned}$$

In the specific implementation of CFAR, given a cell under test (CUT), it is necessary to determine the guard region and the training region, and then estimate the noise level based on the value of the training cells, and thus obtain the detection threshold as follows

18 $$\begin{aligned} V_{T} = q * \sigma _{\varepsilon }, \end{aligned}$$

where *q* is a scaling factor related to $P_{fa}$. By choosing a suitable scaling factor, the false alarm probability can be kept as a proper constant. $\sigma _{n}$ denotes the estimated noise power, For each CUT, the noise power can be obtained by calculating the average value of the training cells as follows.

19 $$\begin{aligned} \sigma _{\varepsilon } = \frac{1}{M} \sum _{m=-M/2}^{M/2} s_{p}(t+m), \end{aligned}$$

where *M* denotes the number of training cells^20–22^.

**Figure 2:**
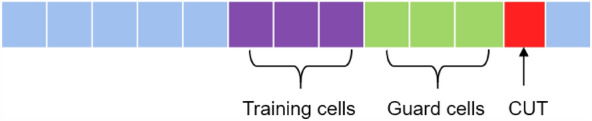
CFAR detection parameters configuration diagram.

It is worthwhile to note that the function of the guard cells is to define a neighborhood around the cell under test (CUT) so that the cells within that neighborhood will not be used for noise level estimation, as shown in Fig. 2. The larger the number of guard cells, the farther the training cells are from the CUT. In practice, the leakage signal is not an ideal impact function but occupies a certain width, in this case, the selection of the guard cells should be at least larger than the width of the leakage signal, otherwise a part of the leakage signal will be used for the noise estimation, thus affecting the setting of the constant false alarm threshold.

The function of the training cells is used for the estimation of the noise level, as shown in Figs. 1 and 2. The noise level is estimated using sample mean, so the accuracy of the noise level estimate depends on the number of training units. Different number of training cells will give noise levels with different estimation accuracy.

The false alarm rate acts as a compromise between the false alarm and detection ability as shown in Fig. 1, i.e., the choice of the threshold determines the false alarm rate setting and the detection probability. If a higher false alarm rate is accepted, then it will also result in a higher detection probability.

The traditional CFAR detection is performed by selecting training cells on the both left and right sides of the CUT^23, 24^. However, in this work, the pipeline leakage occurs instantaneously. To ensure that the response time for detection is as short as possible, the training cells should not use samples after the moment of the CUT, so the left regions of the CUT are used as the training cells. The schematic diagram of the selection of the training unit is shown in Fig. 2.

### Pipeline leakage detection algorithm

**Figure 3:**
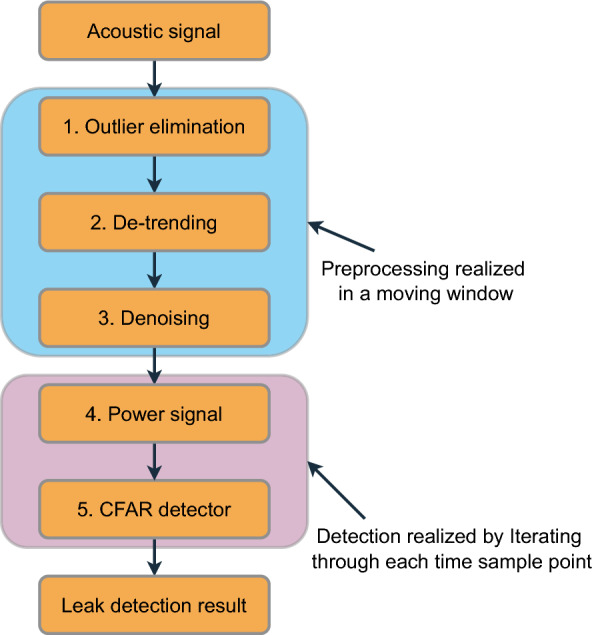
Algorithm flow chart.

Based on the above analysis, the pipeline leakage detection algorithm flow by CFAR is summarized as shown in Fig. 3.

In total, the detection algorithm is divided into two main parts: signal preprocessing and leak incident detection. The algorithm modules are described in detail as follows.

1. Outlier elimination: outliers are usually characterized by randomness and sparsity, so median filtering is used to eliminate them. Here, the time sampling points are processed sequentially using a moving window.
2. De-trending: detrending: processing in a moving window, the first and last points of the signal intercepted by the window function are differenced to achieve the removal of the trend term.
3. Denoising: processing using moving window averaging.
4. Power signal: power signal generation: CFAR detection is based on the power signal, so the power of the acoustic signal needs to be considered to ensure that the negative values in the signal do not affect the detection effect.
5. CFAR detector: finally, the CFAR detector averaged over the left side cells is used to perform the detection of pipeline leakage signals.

It is worth noting that in the actual implementation of the algorithm, the selection of the number of guard and training cells needs to take into account the duration of the acoustic signal at the time of the leakage incident. The general rule is that the guard cells should be able to effectively cover the length of the acoustic signal in order to ensure that the leakage incident signal is not treated as a noise term for noise level estimation. Meanwhile, due to the presence of the training and guard cells, there will be some detection blind areas in the left boundary part of the signal. However, these detection blind areas have been detected in historical time and thus do not affect the detection capability of the real-time system.

## Experimental results and discussion

### The design of real-world simulation experiment

**Figure 4:**
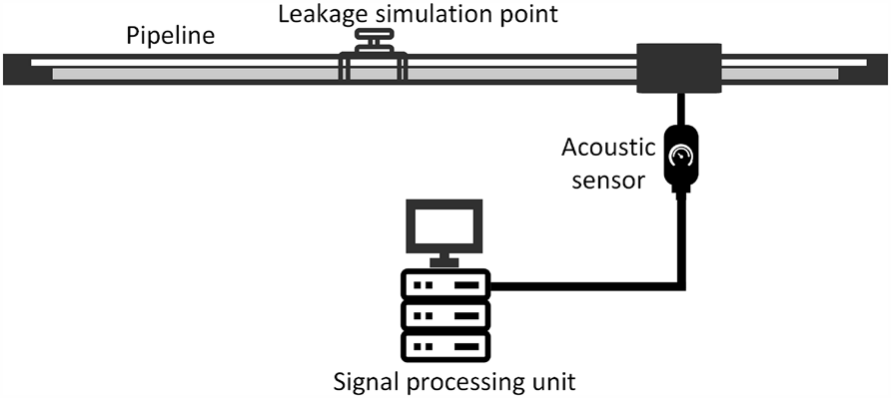
Schematic diagram of the experimental design.

The schematic of the real-world leakage simulation test is shown in Fig. 4. In this experiment, acoustic sensors were installed on the pipeline to collect acoustic signals. An artificial leak point was set up in the middle of the pipeline to simulate a pipeline leak incident. When the valve at the leak point is opened, the transmission medium in the pipeline is released from the leak point, generating a leak acoustic signal that is recorded by the sensors. The data received by the acoustic sensors is converted to a digital signal by an analog-to-digital converter and transmitted to a central computer, which processes the digital signal to determine whether a leak has occurred.

**Figure 5:**
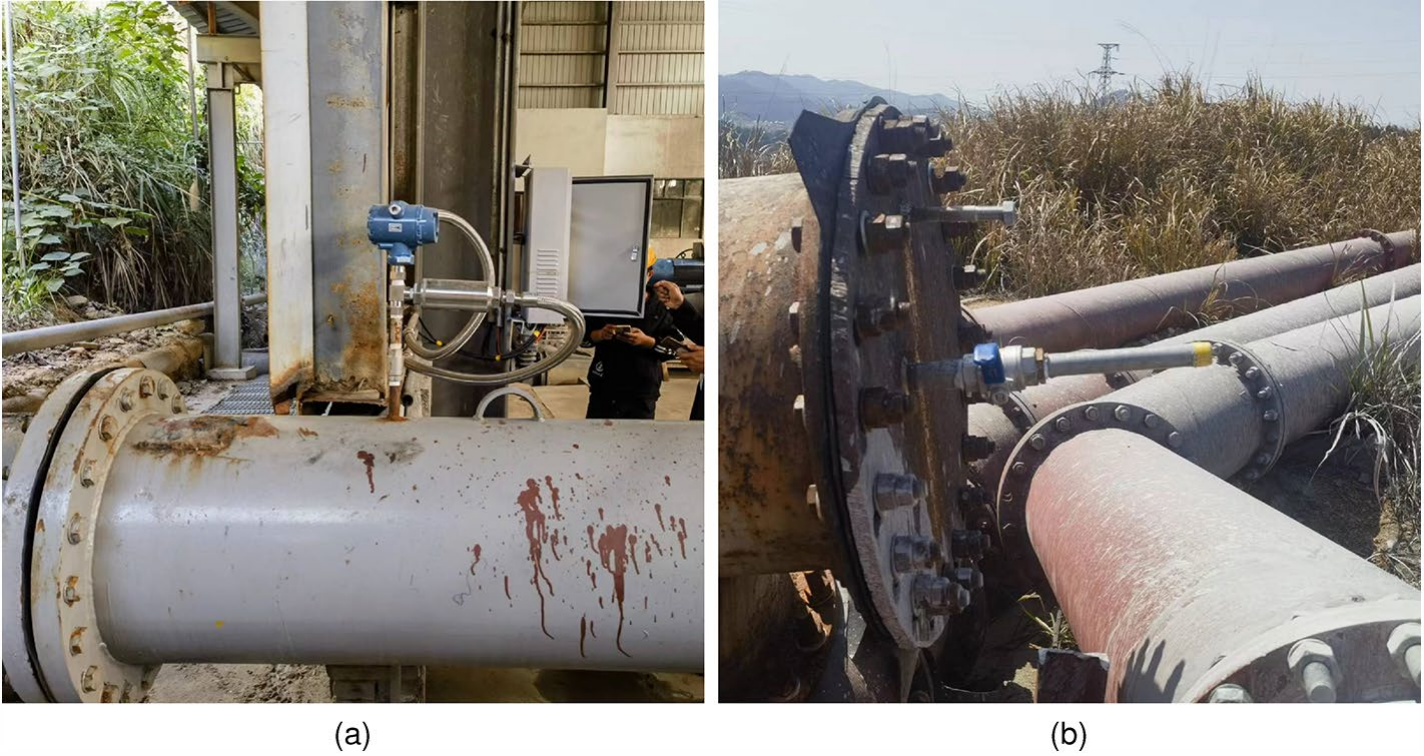
Pipeline leakage simulation test site photos. (**a**) Acoustic sensor installation location; (**b**) artificially simulated leak point location.

**Figure 6:**
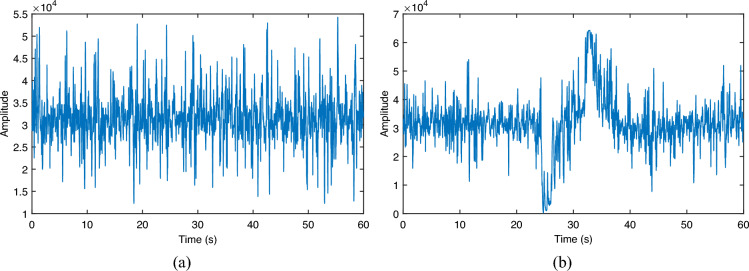
Pipeline signals. (**a**) Normal signal; (**b**) leak signal.

This real data was collected from a simulated leak test of a pipeline. The data collection time was 2 min, and the sampling period of the sensors was set to 10 ms. The total length of the pipeline was 78.3 km, and the experiment was carried out in the form of a simulation test with a fast-opening and fast-closing valve at 49.8 km from the first station of the pipeline to simulate the leak incident with a leak hole diameter of 5 mm, 7 mm, and 10 mm, respectively. The photographs of the experiment site are shown in Fig. 5.

The acoustic signal of a typical pipeline operation is shown in Fig. 6. A digital signal for normal operation and a digital signal for the occurrence of a leakage incident are given, where the vertical coordinate represents the quantized sampling value of the voltage, from which it can be seen that when a leakage occurs, there will be a more distinct change in the signal characteristics.

### Experimental results

#### Signal preprocessing results

**Figure 7:**
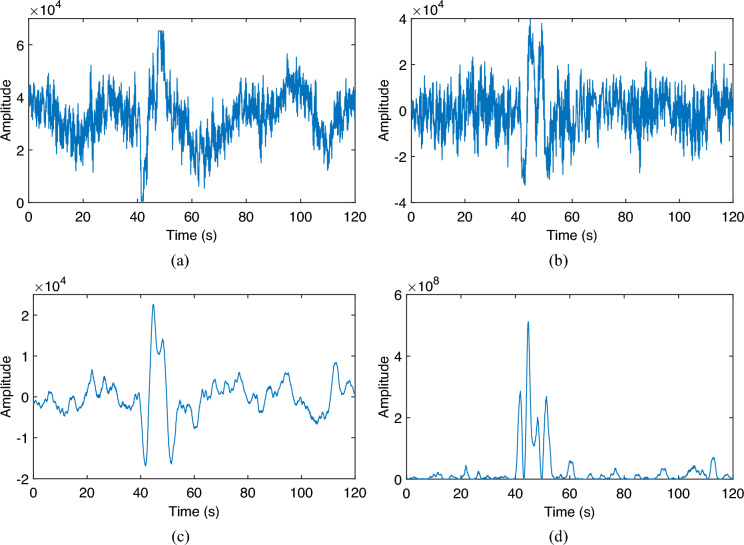
Preprocessing results of leak acoustic signal. (**a**) Original signal; (**b**) difference signal; (**c**) denoised signal; (**d**) power signal.

For the acoustic signals containing leakage events which occurs at 40.8 s, the results after preprocessing are shown in Fig. 7. It can be seen from the original signal shown in Fig. 7a that it contains more obvious trend term and noise. The trend is caused by the change of working conditions in the pipeline. The noise, on the other hand, originates from the thermal noise of the sensor circuit. After the de-trending process, it can be seen from Fig. 7b that the slow trend of the signal is eliminated, while the characteristics of the leakage signal are retained. After further denoising process, the noise in the signal is suppressed and the leakage signal features are more obvious as shown in Fig. 7c. Finally, the power signal is calculated as shown in Fig. 7d.

Comparing the original signal, it can be seen that the preprocessing eliminates the factors in the signal that are detrimental to the detection of leakage events, making the leakage incident signal more significant and making the subsequent detection process more robust.

#### Evaluate the impact of different parameter settings of the CFAR detector

The parameters that need to be set during the implementation of the CFAR detector are the number of guard and training cells, and the false alarm rate. The effectiveness of the detector is related to these parameter settings. The relationship between detection results and CFAR parameter settings is given in this section for a reference during practical implementation.

**Figure 8:**
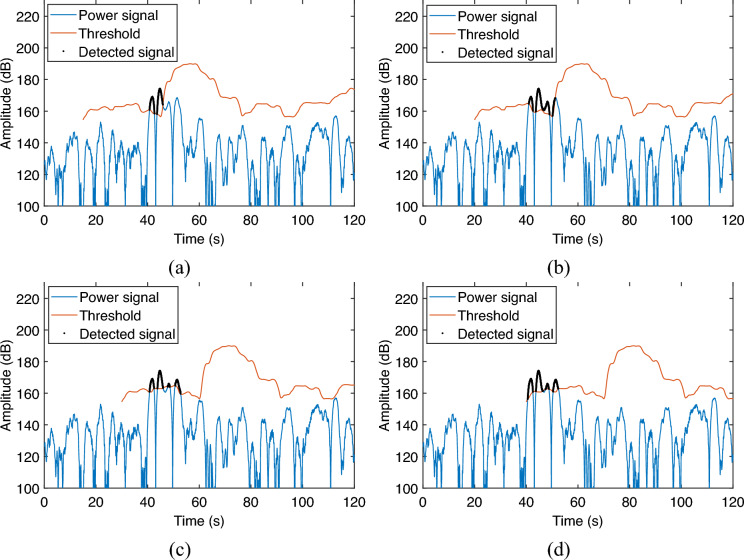
Detection of leak signal in different guard cells setting. (**a**) 5 s; (**b**) 10 s; (**c**) 20 s; (**d**) 30 s.

Firstly, the detection effect is evaluated as the number of guard cells increases. The number of guard and training cells is converted into the length of time based on the sampling interval in order to compare with the actual occurrence time. In this case, the training cells and the false alarm rate are fixed as 10 s and $10^{-16}$ respectively. The guard cells are set to 5 s, 10 s, 20 s, and 30 s, respectively for evaluating the leak detection effect. The results are shown in Fig. 8.

As can be seen, when the guard cells are small, such as set to 5 s and 10 , the length of the guard cells cannot cover the whole segment of the leakage signal, which makes the front part of the leakage signal used as the training cells and thus raises the detection threshold, resulting in the back part of the leakage signal cannot be effectively detected as shown in Fig. 8a,b. When the guard cells are set to 20 s and 30 s, the length of the guard cells is already longer than the 17 s length of the leakage signal, so the whole section of the leakage signal can be effectively detected as shown in Fig. 8c,d. In addition, it is worth noting that when the guard cells are set too long, it will increase the length of the detection blind zone as shown in Fig. 8d. Therefore, in this experiment, the guard cell setting of 20 s is appropriate.

**Figure 9:**
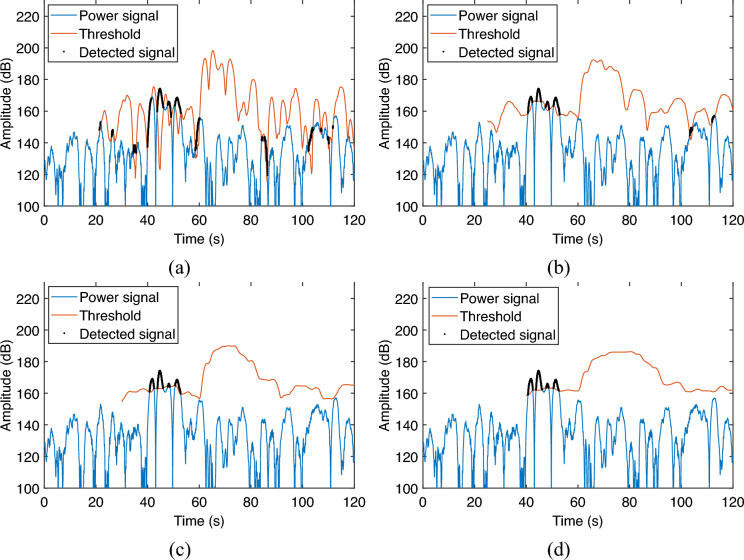
Detection of leak signal in different training cells setting. (**a**) 1 s; (**b**) 5 s; (**c**) 10 s; (**d**) 20 s.

Then, the detection effect is evaluated as the number of training cells increases. In this case, the guard cells and the false alarm rate are fixed as 20 s and $10^{-16}$ respectively. The training cells are set to 1 s, 5 s, 10 s, and 20 s, respectively for evaluating the leak detection effect. The results are shown in Fig. 9.

**Figure 10:**
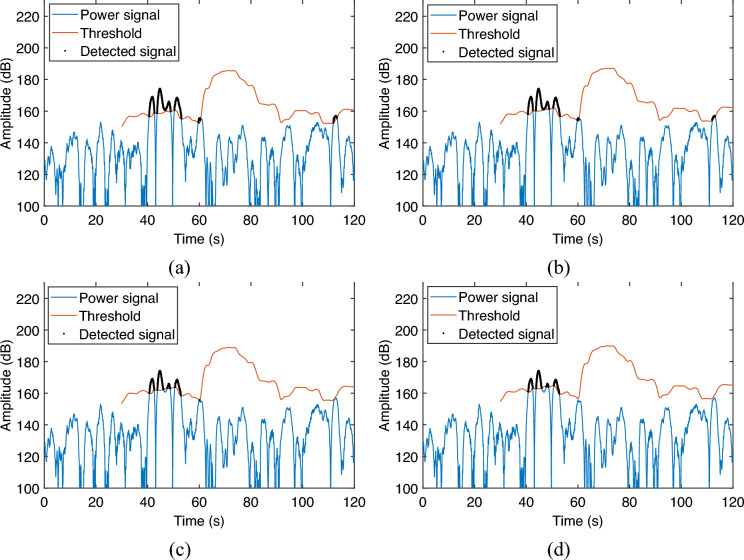
Detection of leak signal in different false alarm rate setting. (**a**) $10^{-6}$; (**b**) $10^{-8}$; (**c**) $10^{-12}$; (**d**) $10^{-16}$.

It can be seen that when the training cells are small, such as the training cells are set to 1 s, only a small amount of training data is involved in the estimation of the noise level, which makes the estimated value of the noise level fluctuating greatly due to the change of the moving window, thus failing to achieve a good detection effect, and even failing to detect valid signals and making false alarms as shown in Fig. 9a. As the length of the training cells increases, the detection effect gradually improves, and when the training cells are set to 10 s and 20 s, the leakage signal can already be detected correctly Fig. 9c,d. It is worth noting that when the training cells are too large, it increases the amount of training data, making the computation time grow, which in turn affects the real-time performance of the system and increases the difficulty and cost of system implementation. Therefore selecting the appropriate training cells is necessary in practice.

Finally, the detection effect is evaluated as the change of the false alarm rate. In this case, the training and guard cells are fixed as 10 s and 20 s, respectively. The false alarm rate is set to $10^{-6}$, $10^{-8}$, $10^{-12}$, and $10^{-16}$, respectively for evaluating the leak detection effect. The results are shown in Fig. 10.

It can be seen that when the false alarm rate is large, it is easy to detect noise as a leakage signal, making the detection results erroneous as shown in Fig. 10a,b. This can be costly in practice in terms of operating costs. As the false alarm rate decreases, the detection threshold is gradually increased so that the leakage signal is detected correctly without false alarms as shown in Fig. 10d. Therefore, in practice, it is necessary to determine the appropriate false alarm.

#### Multiple experimental results and runtime evaluation

The results of the multiple tests are shown in Table 1. A total of 324 simulated leakage tests are conducted to verify the detection accuracy of the proposed detection method.

**Figure 11:**
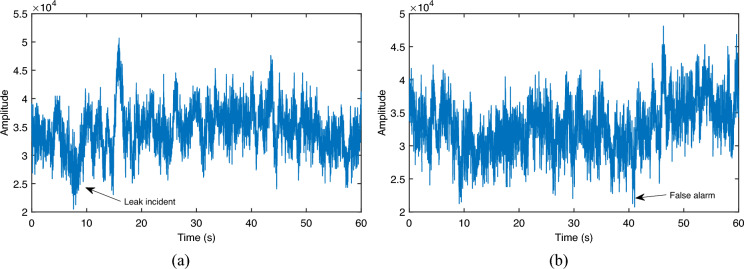
Anomaly detection samples. (**a**) Missed detection; (**b**) false alarm.

There are a total of 91 leakage tests for the 5 mm diameter, 133 leakage tests for the 7 mm diameter, as well as 100 leakage tests for the 10 mm diameter. In the 5 mm diameter test, 77 leaks are successfully detected, 11 leaks are missed, and 3 false alarms occur. In the 7 mm diameter test, 130 leaks are successfully detected, 2 leaks are missed, and 1 false alarm occurs. In the 10 mm diameter test, 98 leaks are successfully detected, 2 leaks are missed, and there are no false alarms. Correct detection rates of 84.6%, 97.7% and 98% are achieved for the three leak diameter settings, respectively. The overall correct rate is 94.1% in all tests.

From the test results, it can be seen that when the leakage diameter decreases, the correct detection rate decreases and false alarms increase. The reason for this is that the SNR of the acoustic signal decreases when a small diameter is leaked, which reduces the difference between the signal distribution and the noise distribution shown in Fig. 1, thus leading to an increase in the false alarm rate and a decrease in the detection probability. A missed detection sample and a false alarm sample in 5 mm diameter are given in Fig. 11. It can be seen that the SNR is low at this point and the signal of the leakage incident is no longer apparent.

The results of the multiple simulation tests demonstrate the effectiveness of the proposed method in real-world scenarios.

**Table 1:** Multiple detection results based on simulation test.

Test index	Leak hole diameter (mm)	Total test number	Number of the detectable	Number of the missed	Number of false alarm	Correct rate (%)	False alarm rate (%)
1	5	91	77	11	3	84.6	3.3
2	7	133	130	2	1	97.7	0.7
3	10	100	98	2	0	98	0
Total	–	324	305	15	4	94.1	1.2

We also evaluate the running time of the proposed method. The runtime of the proposed method is tested using data from one leak test. The computer configuration used in this test is as follows.

- CPU: Intel i5 8300H
- Memory capacity: 8 GB
- Operating system: Windows 11

In this configuration, the time to process one test data, i.e. 2 min length of data is 4 s. With a better test platform, such as an embedded platform, the computation time would be reduced to meet the actual real-time detection requirements.

#### Discussion of multi-leak incident detection

When leaks occur simultaneously at different locations in a pipeline, for a single acoustic sensor, the leak signals generated at different leak locations have different positions in the received signal due to the different arrival times at the sensor. In this case, detection is still possible. In addition, if the distance between different leak locations is far enough to cause the detection signal-to-noise ratio to be too low, it is also possible to consider segmenting the pipeline by distance and installing sensors on different segments to cover the needs of pipeline monitoring over long distances in practice.

## Conclusion

In order to overcome the disadvantages of manual detection such as high cost, poor real-time and low accuracy. An automatic pipeline leak detection algorithm based on acoustic signals is proposed by drawing on the CFAR detection technique in radar target detection theory. Based on the real-world experiment data, the detection effect under different CFAR detector parameters is analyzed, and the results show that the proposed method can obtain a better automatic detection effect under the appropriate CFAR detection parameter settings. The results of multiple experiments verify the effectiveness of the proposed method in practical scenarios.

## Data Availability

The datasets used and/or analysed during the current study available from the corresponding author on reasonable request.
